# Automated Tracking of Drosophila Specimens

**DOI:** 10.3390/s150819369

**Published:** 2015-08-06

**Authors:** Rubén Chao, Germán Macía-Vázquez, Eduardo Zalama, Jaime Gómez-García-Bermejo, José-Ramón Perán

**Affiliations:** 1 University of Valladolid, Paseo del Cauce 59. Valladolid 47011, Spain; E-Mails: chaos.ruben@gmail.com (R.C.); g.macia.vazquez@gmail.com (G.M.-V.); 2 University of Valladolid, Instituto de las Tecnologías Avanzadas de la Producción, Paseo del Cauce 59. Valladolid 47011, Spain; E-Mail: ezalama@eii.uva.es; 3 Fundación Cartif, Parque Tecnológico de Boecillo, Valladolid 47151, Spain; E-Mail: peran@cartif.es

**Keywords:** moving object sensing, computer vision, tracking, prediction methods

## Abstract

The fruit fly *Drosophila Melanogaster* has become a model organism in the study of neurobiology and behavior patterns. The analysis of the way the fly moves and its behavior is of great scientific interest for research on aspects such as drug tolerance, aggression or ageing in humans. In this article, a procedure for detecting, identifying and tracking numerous specimens of Drosophila by means of computer vision-based sensing systems is presented. This procedure allows dynamic information about each specimen to be collected at each moment, and then for its behavior to be quantitatively characterized. The proposed algorithm operates in three main steps: a pre-processing step, a detection and segmentation step, and tracking shape. The pre-processing and segmentation steps allow some limits of the image acquisition system and some visual artifacts (such as shadows and reflections) to be dealt with. The improvements introduced in the tracking step allow the problems corresponding to identity loss and swaps, caused by the interaction between individual flies, to be solved efficiently. Thus, a robust method that compares favorably to other existing methods is obtained.

## 1. Introduction

The *Drosophila Melanogaster* has become a powerful model system for analyzing the relationship between genes, neurons and behavior. Important research efforts carried out with this insect have allowed an understanding of many behaviors of medical interest such as substance abuse [1,2], aggressivity [3,4], sleep deprivation [5], ageing [6] and memory loss [7], among others. The wide range of genetic manipulations in the *Drosophila* makes this animal an ideal genetic system for analyzing the general principles of neuroscience. However, the analysis of the behavioral effects of these manipulations is hampered by the lack of effective methods to measure the flies’ behavior precisely and quantitatively.

In this context, the approaches based on the use of computer vision-based sensors represent a promising strategy for the automated tracking and behavior characterization of the specimens under study. However, the results obtained with these sensors are highly dependent, not only on the quality of the image acquisition system, but also on many other aspects related to the interaction between the insects (overlapping, occlusions, trajectory crossing, collisions) and the presence of eventual optical artifacts (shadows, reflections). All this hinders the robust tracking of the flies through time.

In this paper, a method for dealing with the above-mentioned effects, using an optimized strategy, is proposed in order to achieve an efficient, automated fly tracking.

The paper is organized as follows. The related work is reviewed in Section 2. The proposed algorithm is presented in Section 3. Section 4 shows some experimental results. Finally, concluding remarks are given in Section 5.

## 2. Related Work

The study of the behavior of the *Drosophila* fly and other animals requires detailed observation, annotation and subsequent analysis of the data obtained. The number of individuals may range from a few to a great number (say, hundreds), depending on the experiment. Moreover, the individuals may be studied either alone or in groups, and with or without interaction. Frequently, video material is recorded for an ulterior, off-line analysis of the trajectories, aimed at obtaining confident results.

Specimen observation and annotation is generally carried out by a human operator, which entails frequent errors, given that continued attention is required for long periods of time.

For example, neurobiological research aimed at quantifying the motor decrease of specimens after a genetic modification is shown in [8]. To achieve this objective, specimens were measured at regular intervals, using a chronometer, through time periods ranging from a few hours to several days. This represents a considerable effort and, eventually, the resulting confidence may be compromised. In this context, computer vision sensing is a promising approach for the automated tracking and analysis of the behavior of *Drosophila* and other animals through time.

The tracking of a moving element within a given scene requires a suitable discrimination between this element and the background, *i.e*., a suitable segmentation. Some common segmentation techniques are reviewed in [9,10], while some segmentation techniques based specifically on background modeling are discussed in [11].

Some practical examples of moving object segmentation upon background modeling are: video surveillance [12,13], motion capture [14,15], industrial quality control [16] and the multimedia, entertainment and cinema industry in [17,18].

Roughly speaking, background modeling techniques are aimed at obtaining an image of the scene without the moving objects. This may represent a challenging objective, given that the background appearance may vary through time (due to lighting changes), some elements of the scene may appear/disappear at (or during) a given time, quasi-stationary elements may also be present, *etc.* Different techniques aimed at obtaining robust and adaptive results under these circumstances have been proposed. These can be classified into: basic background models [19,20], statistic background models [11], fuzzy models [21,22], and models based on estimation [23,24]. The models can also be studied in terms of prediction [25], recursiveness [12], adaptability [26], or modality [27], *etc.*

All these techniques share some common features: a background model is first obtained; then the model is initialized and updated throughout the experiment; the foreground is obtained; the elements in the foreground are discriminated on the basis of their size in the image; and the main features of the desired target element are chosen (color, shape, movement, texture, *etc.*).

Once the target element has been detected, it must be tracked through the image sequence. Tracking methods have experienced a considerable advance in recent years, given their interest within the field of automatic video analysis. Some application examples are traffic monitoring [12,13], motion analysis [14,15], human-machine interaction [28], and many others.

A review of tracking techniques can be found in [29,30]. They can be classified according to the way the shape and/or appearance of the objects is represented (points, geometric shape, silhouettes or contours, articulated models, templates, *etc.*) and the features to be tracked (color, shape, optical flow, texture, *etc.*). Silhouettes are used in [31] for tracking objects in a video surveillance application. Objects are represented as ellipses in [32], from which histograms modeling their appearance are obtained. Objects are modeled through rectangles and the corresponding eigenvectors in [33]. Objects are characterized using points that are tracked throughout the image sequence in [34]. A general purpose tracking algorithm that operates on lidar data, instead of images, can be found in [35]. More recently, [36] developed another algorithm that is able to track a group of animals. In this case, several images of a recorded video are used to identify each individual by modeling its appearance.

Concerning *Drosophila*, some representative works are [37,38], where the analysis of the behavior of specimens is addressed, and [39] where a complete analysis of the trajectories is performed. A tracking method for monitoring isolated specimens of *Drosophila* (and other animals) is presented in [40,41,42]; while a method for tracking groups of specimens interacting within a planar scene is presented in [29]. This is one of the most representative methods to date and has been used as the reference in the current work. Other works have focused on the 3D tracking of flies [43], with a high set-up cost, and [44], which uses 3D tracking of luminescent molecules of flies in a vial. Nonetheless, 2D tracking is often preferred, given that it provides similar information at a lower set-up cost. More recently, [45] have proposed a simple image processing algorithm for targeting a single fly with a laser in order to manipulate the fly’s nervous system.

Some of the methods cited above have been used widely, even in commercial systems, but are still open to improvement in some aspects. Concerning [40,41,45] the analysis of individuals interacting with each other within a group is not undertaken. Concerning [29], the tracking error could be improved through a refined tracking strategy, as is proposed in the current work. In addition, that method is highly dependent on the fly plate, which results in a limited flexibility.

With respect to systems that use some of the methods reviewed in this section, it is worth mentioning *Noldus Ethnovision, BioTrack, Idtracker* and *C-Trax*.

*Noldus Ethnovision* [46] is a commercial system able to track several animals moving over a flat environment. The system can operate favorably in the case of large animals (such as mice, rats and others), given that multiple points on each specimen are used for tracking. However, in the case of *Drosophila*, the system cannot deal successfully with some common situations, such as track crossing and specimen occlusions. The system can track multiple individuals only when they do not interact.

*Biotrack* [47], based on [35], is a multi-tracking software that can be used for free. It is a general purpose solution that can track several kinds of animals in a flat arena but deals partly with crossings and occlusions.

*Idtracker* [48] is an open source software that uses an algorithm able to track individuals in a group [36]. Nonetheless, this software cannot make corrections in real time in order to avoid propagating identification errors.

*C-Trax* [49] is a freely available software for *Drosophila* tracking, developed by Caltech (Californian Institute of Technology). This system suffers from eventual tracking errors (loss and swap of individual identities) derived from the selected tracking procedure, which uses a weakly characterized dynamic model, based on [29]. Moreover, the fly plate is shape-restricted and the fly tracking is not performed in real time.

To summarize, the solutions proposed to date for *Drosophila* automated tracking suffer from several drawbacks. Some systems can track isolated flies, which severely limits the analysis of certain behaviors. Other systems can track several flies but cannot hold individual identities when two or more flies approach or overlap. Moreover, some practical restrictions are often present, such as a reduced flexibility to adapt to new experiments, or an inability to operate in real time and, in some cases, an important cost.

In the present work, a new model for *Drosophila* tracking is proposed that allows several interacting specimens to be identified and tracked under noisy conditions and eventual specimen overlapping. The main advantages of the proposed method derive from the proposed tracking method. A tracking strategy based on the optimal Kalman filter, which minimizes the prediction error, thus significantly reducing the identity swaps, is proposed. In previous works, such as [29], obtaining detailed spatial information gained priority over obtaining a dynamic model of the flies. The current approach is also aimed at obtaining detailed spatial information. However, the use of this information is improved significantly by using the Kalman filter, which results in an improved prediction of the system’s state and, therefore, a significant reduction of the error rate (given that individual identities are better preserved though time). Furthermore, the proposed methodology is robust and adaptable: the method does not rely on a correct detection of the fly plate and even fly reflections at the plate boundaries are automatically removed. This confers the system a significant flexibility, given that different plates can be used without requiring any previous adaptation of the system.

## 3. The Algorithm

The algorithm proposed in this paper operates in three main steps: pre-processing, processing and tracking.

### 3.1. Preprocessing

The images are converted into grayscale and processed through a Gaussian filter. Then, the plate is detected and a background model is computed. Finally, a shape model of the specimens is computed for their ulterior detection and tracking.

### 3.2. Plate Detection

The plate is the region of interest where the movement detection will be carried out. In the present work, the plate is detected by using a Canny filter [50] followed by a circle Hough transform [51]. This is appropriate for circle-shaped plates, which is the most common case. Similar procedures could be applied for other plate geometries.

### 3.3. Background Modeling

In general, video processing systems seek to extract moving elements *(foreground) from* stationary elements (background) in the images [9,10]. This can be achieved by computing a proper model of the background.

In our case, the Simple Gaussian method (SG), with selective updating [9], has been used, given the unimodal nature of the background. Small changes of background pixel brightness are modeled through a unimodal Gaussian defined upon estimates of the brightness mean and deviation. In our case, median and median absolute deviation MAD [52] have been used for an increased robustness. A correction term is then applied to fit the correct data into one standard deviation:

(1) $$\text{μ}\left(x,y\right)={\text{med I}}_{\text{t}}\left(x,y\right)$$

(2) $$I\left(x,y\right)=\text{c MAD}=\text{c med}\left({\text{I}}_{t}\left(x,y\right)-\text{med}\left(x,y\right)\right)$$

where *x* and *y* are the pixel coordinates, t ∈{0,Δ,2Δ,…,T} and c=1.4826. This value ensures that the correct fraction of data is within one standard deviation around the median [52].

This provides a background model where the value of each pixel corresponding to the Gaussian determined by μ(*x*,*y*) and σ(*x*,*y*) is obtained. This model is updated periodically during the pre-processing step, so that only those pixels that do not belong to the background are revised. Thus, the background will remain stable for eventual lighting variations and/or image quality deficiencies, thus preventing phantom artifacts derived from static flies to emerge [24]. The update period will depend on the acquisition system quality.

### 3.4. Shape Model

Once the background model is computed, the elements of interest in the images are obtained by subtracting the background (Section 3.2), and their areas $A_{i}\;$ are computed. The process is carried out throughout an image sequence to compute the corresponding mean ${\text{μ}}_{Areas}$ and variance ${\text{σ}}_{Areas}^{2}$,

(3) $${\text{μ}}_{Areas}=\overset{i=N}{\underset{i=1}{\sum }}\frac{A_{i}}{N}$$

(4) $${\text{σ}}_{Areas}^{2}=\sum^{\text{​}}\frac{A_{i}^{2}}{N}-{\text{μ}}_{Areas}^{2}$$

where *N* is the number of elements. These two values will be used to determine whether each detected element corresponds to a fly or not upon a probabilistic criterion based on the element area, thus avoiding false positives.

### 3.5. Processing: Element Detection and Segmentation

The detection, segmentation and validation of the moving elements is addressed in the current step [9,53].

### 3.6. Background Subtraction

The foreground, *i.e.*, the moving objects, is obtained by subtracting the $\left({\text{μ}}_{t},{\text{σ}}_{t}\right)$ background model from the current image,

(5) $$p\left(x,y\right)=\{\begin{array}{c} 255-I_{t}\left(\;p\;\right),\;\;I_{t}\left(\;p\;\right)–\mu \left(\;p\right))>N\;{\text{σ}}_{t}\left(p\right)\\ 0,\;\left((I_{t}\left(\;p\;\right)–\text{μ}\left(\left(p\right)\right)\right)\;\leq 0 \end{array}$$

where *p* are the pixel coordinates, 255 is the maximum value possible for the intensity of a pixel and 0 is the minimum. The value of *N* depends on the quality of the data acquisition system, and *N* σ*_t_*(*p*) is the front detection threshold, for {*N* = 0, 1,2,…*n*}. In our case, initially *N* = 10. The better the system we have, the smaller the required value (because of the lower background noise). This value is increased or decreased (with a hysteresis) during the validation phase to split or merge ellipses.

Those pixels where the difference between the brightness value and the background model is under the front detection threshold are assumed to belong to the foreground (or to the background otherwise).

**Figure 1:**
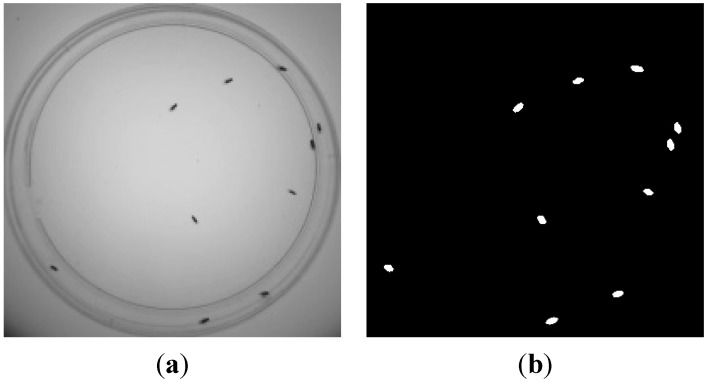
Example of foreground detection. (**a**) Input frame; (**b**) Detected foreground.

An example is shown in Figure 1: the input frame (Figure 1a) and the result after background subtraction (Figure 1b). (Background is displayed in black (0) and foreground in light gray ($255-I_{t}\left(\;p\;\right)$).

It is worth mentioning that images have been acquired using backlighting. Pixels by the center of the flies are darker than the surrounding pixels and the pixel brightness increases as the pixel approaches the fly boundary. This will allow the fitting of the fly brightness by 2D Gaussians, and subsequently, to 2D ellipses.

The elements detected in the image are first classified according to their area,

(6) $$\begin{array}{c} thmin<AREA\;\Rightarrow \;True\\ Otherwise\Rightarrow False \end{array}$$

In this expression, *thmin* is the threshold value that is set to μ*_Areas_* − cσ*_Areas_*, , where ${\text{μ}}_{Areas}$ and ${\text{σ}}_{Areas}$ are the values computed in Equations (3) and (4). This threshold, set to *c* = 15, is used to clean the foreground (in order to obtain a foreground mask free of noise). Moreover, the *c* value selected should be large enough to deal with image noise. In general, the more noise-free the acquisition system is, the larger *c* should be (in our case *c* = 15). It is worthwhile noting that this cleaning process could be done at the validation step, but it is actually done in the processing step to reduce the computing effort of the validation step.

### 3.7. Segmentation

The connected components in the foreground are then fitted to ellipses so that these components can be classified for subsequent tracking [28,31]. The parameters of the ellipses are computed, resulting in a list

**Figure 2:**
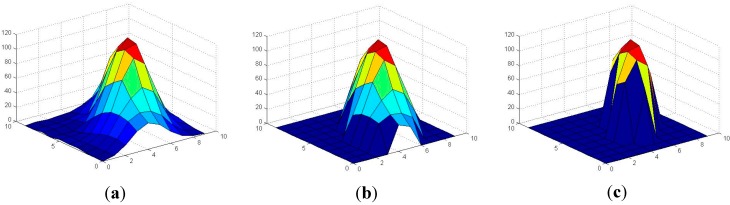
The area of a given fly decreases as the front threshold increases. (**a**) Values in an ROI around a fly (after subtracting the background and computing the absolute value); (**b**) Result corresponding to a threshold value of 50; (**c**) Result corresponding to a threshold value of 70.

(7) Ellipse parameters={(x,y,θ,a,b)}

where *x*, *y*, are the coordinates of the center of each element, θ is the orientation (within ±π), *a* is half the major axis and *b* half the minor axis. *A*, *b* and θ are computed upon the covariance matrix of the Gaussian model, which is obtained from the mean and covariance values, the pixels being weighted by their difference to the background level. Adding weight to each pixel is important because this weight allows connected components to be split in the validation phase: the front detection threshold is increased in a Region Of Interest (ROI) around the fly; therefore, the area of the fly decreases. This can be appreciated in Figure 2, where the levels of a given ROI around a fly (after subtracting the background and computing the absolute value) and the results corresponding to two threshold values, 50 and 70, are shown. The area decreases significantly as the threshold increases.

In this way, if there are two flies close to each other, initially detected as a single object, this object can be split into the two flies by means of increasing the foreground threshold in the validation phase.

(8) Wi=| I(pi)−μ(pi)|/σ(pi)

(9) $$Z=\overset{\;}{\underset{i}{\sum }}Wi$$

(10) $$\text{μ}=\frac{1}{Z}\overset{\;}{\underset{i}{\sum }}Wi\;Pi$$

In this expression {p1,p2, ….pi} are the coordinates of the pixels of a given connected element within the image; Wi is the normalized difference from I(pi) to the average brightness of this pixel within the background model, μ(pi); *Z* is the sum of these differences; and μ their average value. This allows the covariance matrix,

(11) $$\sum^{\text{​}}=\frac{1}{Z}\overset{\;}{\underset{i}{\sum }}Wi\left(\;Pi-\text{µ}\right){\left(Pi-\text{µ}\right)}^{T}$$

to be obtained, which, in turn, corresponds to

(12) $$\sum^{\text{​}}=\;R^{T}{\left(\begin{array}{cc} \frac{a}{2} & 0\\ 0 & \frac{b}{2} \end{array}\right)}^{2}R$$

where

(13) $$R=\;\left(\begin{array}{cc} cos\text{θ} & sin\text{θ}\\ -sin\text{θ} & cos\text{θ} \end{array}\right)$$

The eigen decomposition $\sum^{\text{​}}=U^{T}DU$ provides *a* and *b*, and θ is obtained from *R*:

(14) $$a=2\sqrt{D_{11}}$$

(15) $$b=2\sqrt{D_{22}}$$

(16) θ=arctg(sinθ/cosθ)

The ellipse center location (x,y) corresponds to the central pic of the Gaussian distribution.

### 3.8. Validation

In this step, undesirable artifacts in the images are filtered. In particular, weakly connected components (derived from nearby flies) will be split into the corresponding specimens and spurious artifacts will be removed.

The problem is formulated in terms of a probabilistic model: the fly locations that best explain the current frame are searched. In particular, the set of ellipse locations that maximizes

(17) p(X|I) α p(X) p(I|X)

is searched, where *X* is the said set and *I* is the actual frame.

In general, the location of the different flies may be assumed to be independent, which leads to the formulation

(18) $$p\left(X\right)=\overset{N}{\underset{i=1}{\prod }}p\left(x_{i}\right)$$

(19) $$p\left(x_{i}\right)=e^{\left\{-\left|\text{π}a_{i}b_{i}b-{\text{ μ}}_{Areas}\right|/{\text{σ}}_{Areas}\right\}}$$

where $p\left(x_{i}\right)$ is the likelihood term associated to a normal distribution model of each fly *i*, for *i* = {1, 2, 3...*N*} (*N* being the total number of flies), ${\text{μ}}_{Areas}$ and ${\text{σ}}_{Areas}$ are the shape model parameters, and $\text{π}a_{i}b_{i}$ is the area of the ellipse corresponding to fly *i*.

Finding an analytical solution to this problem would be impractical because p(*X*│*I*) has local maxima and *X* is a discrete set. Therefore, a heuristic strategy has been used. When a connected component has a weak probability, the number of ellipses is increased or decreased to obtain an improved probability.

More specifically, in order to find the connected components corresponding to a given fly, the ellipse enclosing this fly to a large probability (in relation to $p\left(x_{i}\right)$) has to be computed. This probability may be low because the area is either too large or too small. If the area is too large, this connected component may correspond to several nearby specimens. On the contrary, if the area is too small, this component would correspond to a spurious artifact. The foreground detection threshold will then be increased, in the former case, or decreased in the latter. The new elements appearing after this threshold tuning will be assumed to correspond to actual flies if their $p\left(x_{i}\right)$ is high enough, or to a spurious artifact otherwise.

It is worthwhile noting that the number of flies can either be introduced by the user or established by the system automatically in the pre-processing step. If the value is established by the system, it can change if another fly is detected in the tracking phase. In any case, we seek to maximize the probability of P(*X*/*I*) by maximizing the probability of $p\left(x_{i}\right)$ in the validation phase. Summarizing, knowing the number of flies can be useful but is not required by the algorithm.

**Figure 3:**
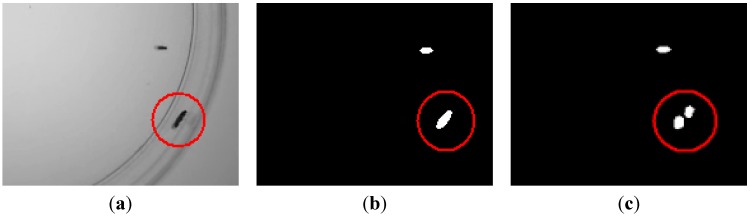
Example of the validation process. (**a**) Input frame, where two flies are in touch (encircled in red); (**b**) Elements found before validation; (**c**) Elements after validation, where the two flies have been properly separated.

An example of the validation process is shown in Figure 3. The detected element in Figure 3b corresponds to two actual flies, as can be seen in Figure 3c.

### 3.9. Reflections

**Figure 4:**
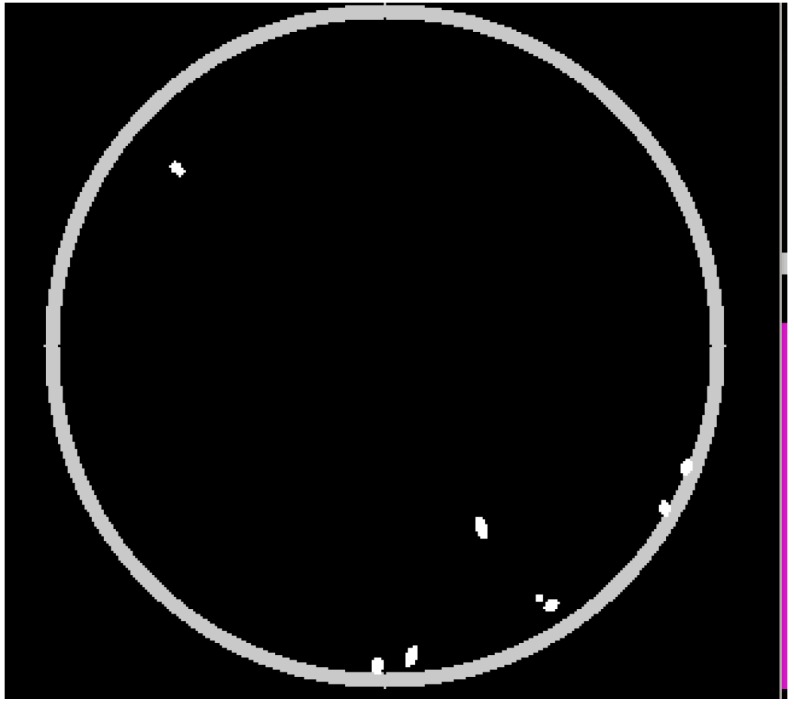
Mask for dealing with reflections at the plate boundary.

The plate border may be highly reflective. Therefore, a method for avoiding spurious elements corresponding to reflections (that would be tracked in an ulterior step) should be used. In our case, a circumference-shaped mask at the plate boundary has been used (*see* Figure 4). Elements entirely within this circumference are masked out.

It is worth noting that the plate may not be accurately detected in some situations. In fact, some image acquisition conditions may hinder this detection. In the current work, the automatic detection of the plate may be manually disabled (when the detection accuracy is not satisfactory). In spite of this, the proposed algorithm can deal with reflections in an ulterior step, as will be discussed in Section 4.3.

### 3.10. Tracking Algorithm

The tracking process is aimed at keeping the flies’ identities throughout the image sequence. Each fly is assigned an identity that should be preserved during the experiment. To this end, deterministic approaches could be used, but these approaches are largely sensitive to noise and other circumstances that may result in the loss of some fly identities (e.g., fly overlap or fusion). In the present work, a probabilistic approach has been used that employs a dynamic model for predicting the position of every fly at time *t* from the current information at *t-*1, using a prediction-correction Kalman filter. This results in an increased robustness against identity losses or swaps.

### 3.11. Kalman Filter

The Kalman filter [54,55] is an optimal, recursive algorithm that uses a stochastic dynamic model of normal-distributed variables and the Bayes theorem. An estimation of the current position is computed upon information from previous states and then refined in such a way that the expected error is minimized.

In the present work, a Kalman filter is started at any new element occurrence in the image sequence. The filter will allow the element location to be predicted, along with the corresponding uncertainty. Then, the real location is searched within a region around the predicted location, according to the said uncertainty.

The approach has been implemented through what we have called a tracker. A tracker is an object that contains all the data concerning the element location and shape, and the parameters and equations related to the Kalman filter. Each validated element is assigned a tracker.

The filter is defined by

(20) $$X_{t}=\left(\begin{array}{c} x\\ y\\ Vx\\ Vy\\ \theta \\ w \end{array}\right)$$

(21) $$A=\left(\begin{array}{cccccc} 1 & 0 & \text{d}t & 0 & 0 & 0\\ 0 & 1 & 0 & \text{d}t & 0 & 0\\ 0 & 0 & 1 & 0 & 0 & 0\\ 0 & 0 & 0 & 1 & 0 & 0\\ 0 & 0 & 0 & 0 & 1 & \text{d}t\\ 0 & 0 & 0 & 0 & 0 & 1 \end{array}\right)$$

where $X_{t}$ is the state vector at time *t*; x, y are the element position coordinates; Vx, Vy  are the linear velocity components; θ is the orientation (within ±π); and *w* is the angular velocity.

*A* is the transition matrix that relates the previous state, at *t* − 1, to the current state, at *t*, subjected to process noise ${\text{ω}}_{t-1}$:

(22) $$X_{t}=AX_{t-1}+{\text{ω}}_{t-1}$$

The state predicted in this way is updated upon the vector of measures $Z_{t}$,

(23) $$Z_{t}=\left(\begin{array}{c} Z_{x}\\ Z_{y}\\ Z_{Vx}\\ Z_{Vy}\\ Z_{\theta }\\ Z_{w} \end{array}\right)=HX_{t}+v_{t}$$

This vector combines the measures of the selected state variables, $Z_{x}$ $Z_{y}$ is the measured position, $Z_{Vx}$ $Z_{Vy}$ is the element velocity, $Z_{\text{θ}}$ is the element orientation (with respect to the previous one; ranged within ±π), and $Z_{w}$ is the angular velocity. Moreover, *H* is the 5 × 5 identity matrix that relates the state to the prediction, and $\;v_{t}$ is the measuring error. ${\text{ω}}_{t}$ and $v_{t}$ are assumed to be independent, Gaussian and zero-centered.

The angular and linear velocities that feed the Kalman filter are not computed by directly deriving the element position and orientation (which could lead to large errors, especially when low image resolution or frame rate is used). Instead of this, an algorithm is used to smooth the data before feeding it to the filter. This algorithm uses a moving average filter with a weight term. The larger the error is, the larger the correction provided by the algorithm will be.

**Figure 5:**
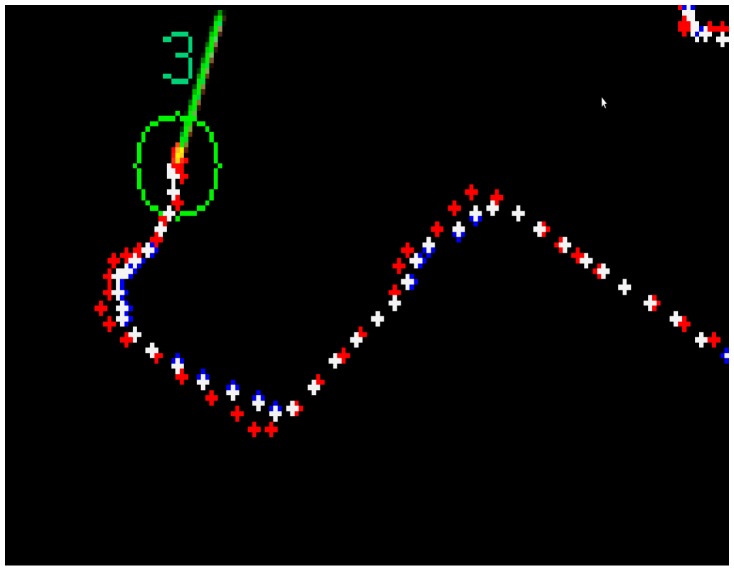
Trajectories generated by a tracker (corresponding to fly number 3 in this example) after several prediction cycles. The measured trajectory is displayed in blue; the predicted one in red; and the corrected one in white.

In a first step, the current state is predicted using all previous information. In a second step, the state prediction is corrected using the actual data measured by the system so that the error is minimized. The prediction-correction depends on the difference between the predicted state and the measured one and on the filter gain, which, in turn, depends mainly on the measure uncertainty [56]. Figure 5 shows an example where measured, predicted and corrected positions are displayed for a given trajectory.

### 3.12. Identity Assignments

The element locations predicted by the Kalman filter feed the identity assignment stage. This stage is a maximization and minimization problem. Many algorithms can be used for solving this kind of problems [57]. In the current work, the Hungarian optimization method, which allows the assignment problems to be solved in $O\left(n^{3}\right)$ time, has been selected. The assignation problem is modeled through an *n* × *n* cost matrix, where the costs are

(24) $$Cost\;=\frac{1}{2{\text{πσ}}_{x}{\text{σ}}_{y}}\;e^{\left[-\;\frac{1}{2}\left(\frac{x-{\text{μ}}_{x}}{\sigma_{x}}\right)\;-\;\frac{1}{2}\left(\frac{y-{\text{μ}}_{y}}{\sigma_{y}}\right)\right]}$$

In this expression, ${\text{μ}}_{x}$, μ*_y_* are the element coordinates predicted by the Kalman filter at *t −* 1; *x*, *y* are the actual element coordinates at *t*; and ${\text{σ}}_{x}$, ${\text{σ}}_{y}$ are the prediction errors of *x*, *y*. Thus, the probability that a given element at frame *t* corresponds to a given element at *t −* 1 is computed and the Hungarian algorithm provides the identity assignments. It is a maximization problem, and each fly will be assigned the tracker corresponding to the highest probability.

Usually, the described algorithm leads to a unique assignment: each element in the image is assigned a single tracker that will store the element’s properties for the next algorithm’s progress. However, eventually, more than one tracker may be assigned to a given element (there are fewer elements than trackers). This happens when two or more elements have merged or overlapped. In this case, the procedure below is used.

### 3.13. Tracking

When an element in the foreground has been assigned more than one tracker, a further segmentation of this element into as many sub-elements as assignments is first explored. This segmentation is carried out upon the image information at time *t*.

In concrete, a validation algorithm is applied, using a threshold value higher than that of the segmentation phase, in order to split the connected component (if possible). The algorithm knows that there are two flies in this area and tries to find them. If the flies do not appear by means of the validation algorithm, then the Kalman filter gives an optimal estimation of the position to the tracker.

If the fly follows a path (does not stop), then it will be found by the tracker by means of the Kalman filter estimation. To be precise, when the assignment is unique, the measure uncertainty is small and the tracker weights the current measure to a higher value than the Kalman prediction. On the contrary, if the assignment is not unique, the uncertainty becomes large and the tracker weights the locations provided by the Kalman filter to a higher value than the current measure.

If the fly being tracked, and another fly (or flies) of the cluster, stops and cannot be split by the validation algorithm, the tracker waits for the fly to appear and the assignment is carried out upon the last known position using the k-means and the maximization expectation algorithms. This strategy, along with the information coming from the other trackers, allows the correct assignment to be established.

When a fly being tracked disappears, the tracker initially uses the information provided by the Kalman filter to find it. If the fly does not appear after a number of frames, the tracker will be waiting for the fly to appear at the last known position, during a given time. (If the fly was a newly detected object, its tracker is simply removed). If other trackers have not missed their flies, then the fly is assigned to the tracker when it reappears. Otherwise, if any other tracker has missed its fly, the fly that has appeared is assigned to a close tracker in the identity assignation phase (by the expectation maximization algorithm).

The procedure is completed by some heuristic strategies that are applied to particular cases. In sum, each time a new tracker is generated (a new element is detected), it is removed if the corresponding element either remains static in the image for a long time (e.g., some plate features detected as elements) or it disappears in the next frames (it was a spurious element or a reflection). Similar strategies are used for the case of a fly moving near the plate boundary, thus disappearing and appearing in close frames, and for reflections that may happen near the plate boundary.

**Figure 6:**
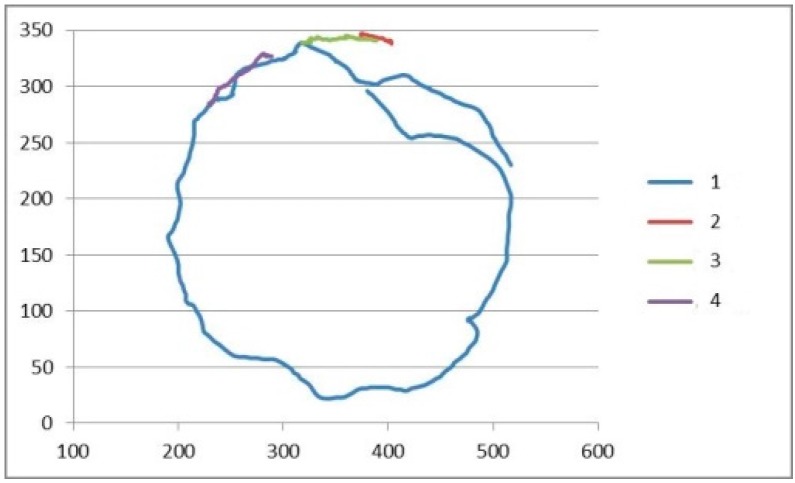
Example of trajectories corresponding to four flies (colored in blue, red, green and purple).

An example is shown in Figure 6, where four flies have been tracked for a given time period. The trackers hold their corresponding element without suffering from undesired identity changes.

## 4. Results and Discussion

In this section, a number of sequences are analyzed that show the behavior of the proposed approach under different situations: usual situations, overlaps, fusions, reflections and poor image quality.

### 4.1. Acquisition System

The proposed method is robust for common situations and for sensing hardware. Image sequences have been obtained using a simple image acquisition system equipped with a common webcam and a LED light spot. A circle-shaped region of the images (that does not necessarily correspond exactly to the plate) is processed. Video sequences have been recorded in a common laboratory, during the day, under daylight conditions. Videos are affected by significant noises of different natures. Sequences of 5000 frames have been acquired and processed.

**Figure 7:**
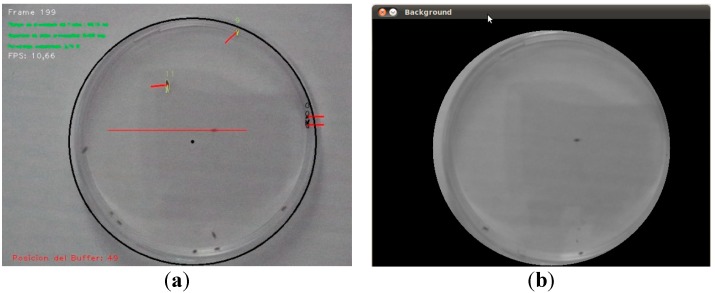
(**a**) Plate under front-lighting, without ambient light screening (the colored lines will be explained later); (**b**) Obtained background model.

**Figure 8:**
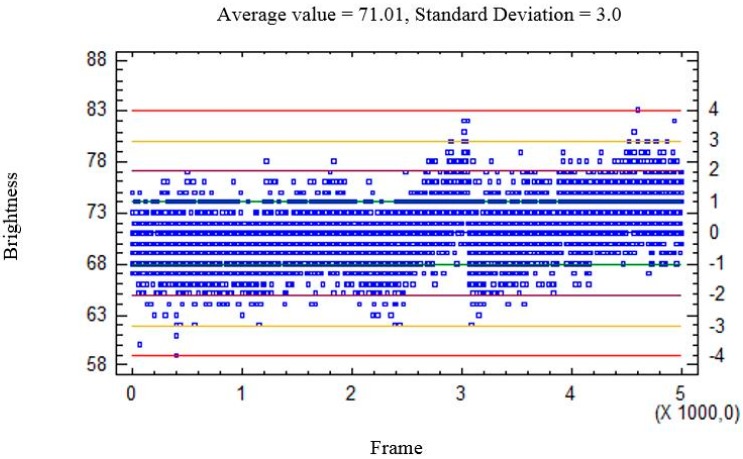
Brightness of a single background pixel against the frame number, in the presence of ambient light. (The pixel is located at the midpoint of the red line displayed by the plate center in Figure 7.) ±*N* σ lines (for *N* = 0, 1,2,3,4) have been also plotted, in blue, green, garnet, yellow and red, respectively. It can be seen that ambient light hampers obtaining a precise background model.

Ambient light makes obtaining a precise background model more difficult, given that the sensed brightness varies within a wide range. An example is shown in Figure 7 and Figure 8. Figure 7 shows the plate under front-lighting (without ambient light screening) and the obtained background model. This model suffers from a large variation in brightness which hinders the detection of moving elements. This variation can be seen in Figure 8, where the brightness of a background pixel (located at the center of the red line in Figure 7a) has been plotted against the frame number (within 1 to 5000). N σ lines have also been drawn (for *N* = 0,1,2,3,4).

**Figure 9:**
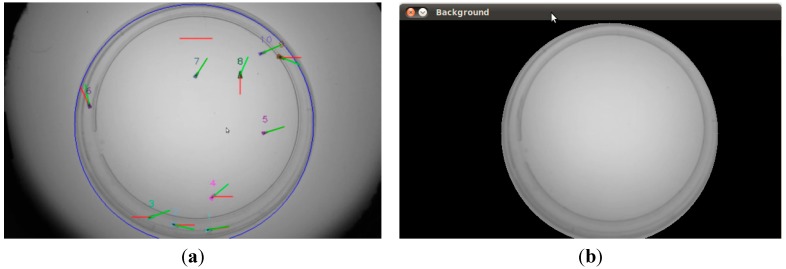
(**a**) Plate under back-lighting with ambient light screening (the colored lines will be explained later); (**b**) Background model.

**Figure 10:**
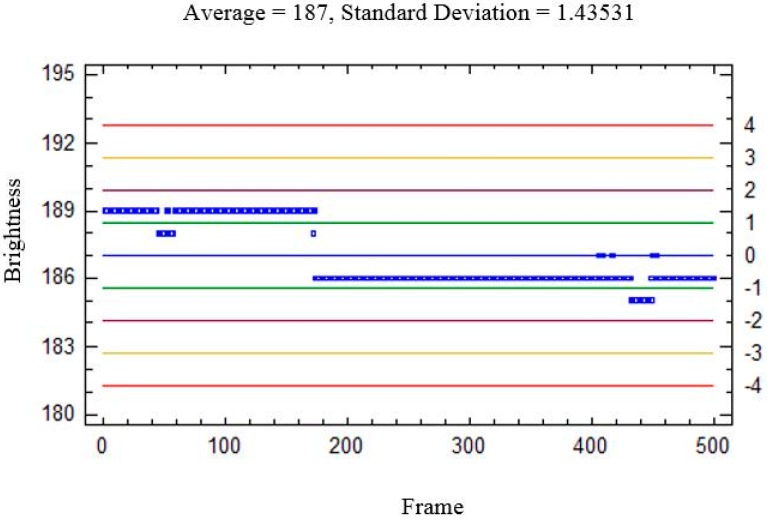
Brightness of a background pixel against the frame number, without ambient light. (The pixel is located at the midpoint of the red line in Figure 9, and ± *N* σ lines have also been plotted.) A precise background model can be obtained in this case.

The result obtained using a back-lighting scheme with ambient light screening is shown in Figure 9. The corresponding brightness variation has been plotted in Figure 10 (in this case, for a 500 frame sequence).

In this case, a precise background model can be obtained, thus allowing a good segmentation of the flies at a lower computational effort (given that a low-frequency background model update will be required). Therefore, this sensing set-up has been selected for subsequent experiments.

### 4.2. Specimen Crossing and Collisions

**Figure 11:**
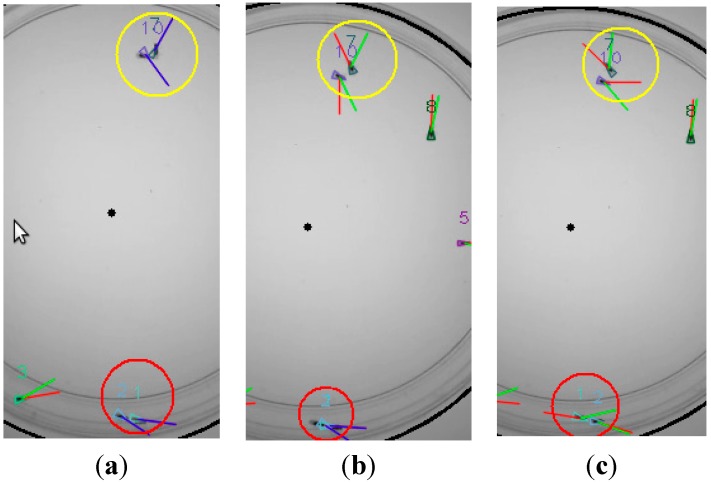
Tracking fly interactions (encircled in yellow and red). Frames at *t*, *t* + 1 and *t* + 2 are shown in (**a**–**c**), respectively. The instantaneous speeds, their moving average and the Kalman filter outputs are displayed red, blue and green, respectively. The identity of the flies is preserved after the interactions thanks to the optimal filter combined with the Hungarian.

Figure 11 shows the behavior of the system when two specimens merge. (Each fly has been labelled a different number and color.) Flies 1 and 2 (encircled in red) and flies 7 and 10 (encircled in yellow) are close to each other at *t* (Figure 11a), so the corresponding blobs merge. Flies 1 and 2 are going to overlap and flies 7 and 10 have already overlapped. The instantaneous speeds, their moving average and the Kalman filter outputs are displayed in red, blue and green, respectively.

In the next frame (Figure 11b), at *t* + 1, flies 1 and 2 are overlapped and flies 7 and 10 have already separated. Finally, in the last frame (Figure 11c), at *t* + 2, the two fly couples have separated completely. It can be seen that the Kalman filter combined with the Hungarian prevent fly identities from being lost or swapped during the whole sequence, *i.e.*, the flies are tracked correctly, as expected.

### 4.3. Reflections

Heuristic strategies have been incorporated to the general procedure to deal with some special situations that may occasionally happen.

**Figure 12:**
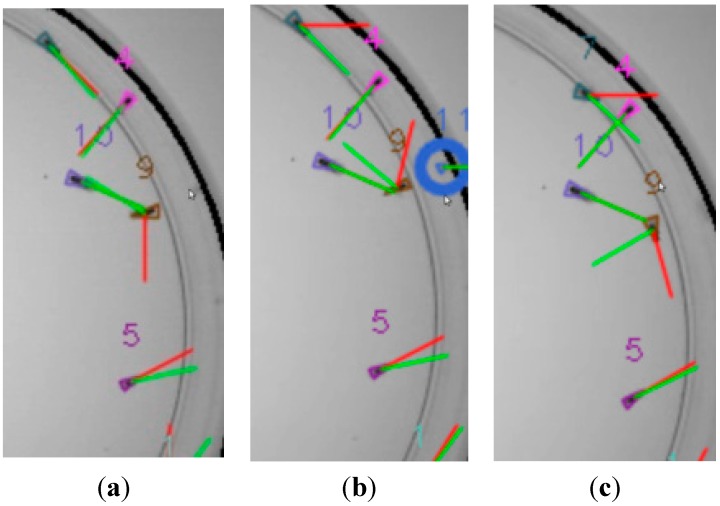
Fly reflection at the plate boundary. (The instantaneous speeds, their moving average and the Kalman filter outputs are displayed red, blue and green, respectively.) Frames at *t*, *t* + 1 and *t* + 2, in (**a**–**c**) respectively, show that the fly reflection (encircled in blue) is correctly removed by the algorithm.

An example is shown in Figure 12. As mentioned in a previous section, the automatic plate detection implemented in this work can be manually disabled when an accurate detection is not possible. Unfortunately, some undesired reflections may appear in this case at the plate boundary. Fly 9 is near the boundary at frame *t* (Figure 12a). A fly reflection artifact that has not been removed in the pre-processing step appears in the image, at frame *t* + 1 (encircled in blue, in Figure 12b). This artifact is assigned a new tracker, tracker 11. Each time a new tracker becomes active, its stability is tested during a few frames (say 50 frames for a 15 fps rate). Then, undesired situations are detected, such as: the tracker loses its fly or the tracker is assigned a fly that has already been assigned another tracker. In this example, the new tracker has been active for too short a time. Therefore, it has been removed automatically and is no longer considered in subsequent frames (Figure 12c). The same procedure can deal with similar situations, such as spurious artifacts caused by poor image quality.

### 4.4. Quantitative Results

The results corresponding to six videos of eight *Drosophila* interacting within a Petri plate, sized at 90 mm in diameter and 6mm in height, are reported. The average fly population was 0.12 flies/cm^2^. The videos were recorded in the afternoon, coinciding with the peak activity of the flies, at a temperature of 28 °C.

Some situations were tested during the experiments, such as a plate being placed partially outside the image or fly food being present in the images. Moreover, different error rates were quantified. The obtained results are reported in Table 1. The experimental conditions are described in Table 2 (along with the conditions corresponding to another method taken as reference).

**Table 1:** Tracking error corresponding to six test videos. The first column shows the video id. The second and third columns show the total number of frames and the number of frames with two or more flies close to each other (occlusion) reported by the software, respectively. Columns 4 to 6 show the number of errors. The last columns report the errors with respect to occlusions and fly density (and time), respectively.

Video	Number of Frames		Errors			Error Frequency	
	Total	With Occlusion	Swap	Lost	Spurious	Error/Occlusions (%)	Error/ρ/t (%)
1	4095	1782	0	1	0	0.84	3.05
2	5025	2181	2	0	0	1.37	4.97
3	5012	1533	2	0	0	1.95	4.98
4	5022	2366	1	0	0	0.63	2.48
5	5044	1834	0	0	0	0	0
6	5005	2024	3	0	0	2.2	7.4

**Table 2:** Comparison between the sensing systems used in the experiments.

	(Current Work)	Reference [29]
Lighting	Backlight LED 800 lumens (with ambient light screening)	Cenital LED 480 lumens (with ambient light screening)
Optics	1280 × 720 Microsoft^®^ LifeCam Cinema™	1280 × 1024-pixel, firewire camera (Basler A622f) equipped with an 8 mm lens (PENTAX)
Resolution	4 pix/mm	4 pix/mm
FPS	15	20
Plate radius	4.5 cm	12.5 cm
Videos	1, 2, 3, 4, 5	*i*, *ii*
Total population (N)	8	50
Population density (ρ)	0.12 flies/cm^2^	0.106 flies/cm^2^

The video id number and the number of frames are annotated in the first and second columns of Table 1, respectively. The number of frames with occlusion (reported by the software), *i.e.*, the number of frames in which two or more flies are merged in a single blob, is shown in column 3.

The tracking errors are reported in columns 4, 5 and 6. Column 4 refers to identity swaps; column 5 to identity losses; and column 6 to spurious artifacts not removed by the algorithm. These figures have been determined manually by watching the processing results.

The last two columns refer to the identity swaps with respect to the number of fly interactions and the number of errors (identity losses plus identity swaps) with respect to the fly population density and the video length (in seconds), respectively. Both results are given in %.

It can be seen that the system behaves suitably concerning fly occlusions and identity losses. In sum, the worst case corresponds to video sequence 6 (the last row in Table 1). This is a 5005-frame video sequence and there are 2024 frames with overlapping situations. However, only three overlapping situations have resulted in identity swaps. Moreover, there has been no identity loss.

The proposed method has also been compared to a state of the art one. The work [29] has been selected, given that it is considered a reference paper on *Drosophila* tracking. Quantitative comparison is a challenging issue, given that the sensing systems used in that work are different from ours. Moreover, environmental conditions such as temperature, time of day, humidity, *etc*., significantly influence the behavior of the flies. Therefore, the variability from one experiment to the other may be large.

Assuming the said variability, the obtained results are reported in Table 3 and Table 4. Table 3 shows the processing time (in frames), the number of occlusions and the error rates corresponding to two video sequences taken from the supplementary Table 1 of [29]. In concrete, we have taken the videos presented in rows 5 and 6 (labeled *i* and *ii* in the present work). These videos have been selected because their fly density is similar to ours (while the fly density in the other videos of that work is lower). It is worthwhile noting that the fly density is a cardinal aspect, given that the larger this density is, the more interactions there will be between flies. Moreover, the error rates have been computed in a different way with respect to the said paper: Error/Occlusions has been computed by considering both the number of losses and swaps (given that, in our opinion, an identity loss is also a tracking error and should be considered as such); and the error in the last column has been computed with respect to the fly population density instead of the number of flies used in the reference method. What is more, we have computed the error using the time (in seconds) because the frame rate is different in the compared experiments. In fact, this frame rate is lower in our case, which results in more tracking difficulty. Finally, we have reported the error rate in %, which is an easily understandable measure.

**Table 3:** Error frequency. Figures have been estimated from the data shown in the reference paper, according to the criteria described in the current section.

Supplementary Table 1 [29]	Number of Frames		Errors			Error Frequency	
Video	Total	With Occlusion	Swap	Lost	Spurious	Swap/Occlusions (%)	Error/ρ/t (%)
*i*	6123	2135	0	2	0	1.87	6.16
*ii*	6587	4545	1	0	0	0.44	3.08

**Table 4:** Comparison between the error frequency for the two methods.

Video	t(s)		Error (Swap + Lost)	Error Frequency	
	Total	with Occlusion		Error/Occlusions (%)	Error/ρ/t (%)
*i*, *ii* [29]	6355	334	3	0.898	4.4
1,2.3,4,5,6 (Proposed method)	1946	984	8	0.813	3.4

Table 4 shows a quantitative comparison between the two methods for a number of experiments (using the error rates defined above). It can be seen that the error frequency with respect to the number of occlusions is similar for both methods. This error quantifies the behavior of the pre-processing step.

However, the error frequency with respect to the population density and the time is clearly lower in the case of the proposed method. This error quantifies the behavior of the fly tracking. Therefore, from the obtained results, we can deduce that the use of the Kalman filter and the other improvements proposed in the current paper result in significant progress.

Some execution examples of the proposed method, operating in real time, can be downloaded at [58]. Some further material (source code, software libraries, demos and documentation) can be downloaded at [59].

A valuable feature of this method is that it can work in real time. Processing takes only about 7 to 15 ms per frame in a (modest) Intel Pentium 4, 3 GHz computer. Moreover, the algorithm is designed in such a way that the pre-processing phase and the tracking phase could be executed in parallel in a dual core architecture.

## 5. Conclusions

In this paper, an automatic system for tracking *Drosophila melanogaster* movements has been presented. The proposed method deals efficiently with problems derived from limited image quality and the interaction among flies.

The limited image quality results in the modification of the aspect of the flies that, in turn, hinders their detection and would cause identity losses during tracking. The described pre-processing and processing steps deal suitably with these problems and allow an accurate foreground to be obtained with low computational effort. Shadows, reflections and phantoms are also avoided. Thus, the cost of the required system hardware is kept low as far as both the acquisition system and the computing resources are concerned.

Moreover, the proposed tracking algorithm allows an accurate prediction of the system state to be obtained with respect to other existing algorithms, thus resulting in a significantly reduced rate of fly identity losses and swaps. Moreover, the method has been completed with some heuristics that deal with problems that may eventually happen, such as spurious artifacts and fly reflections at the plate border. Furthermore, the correct tracking does not require an accurate detection of the plate, which eases the method adaptation to different experiments. The whole procedure—pre-processing, processing and tracking real time—can operate in real time.

The method allows detailed and precise information on the flies to be obtained concerning both their dynamics and their behavior. The distance travelled by each fly, the instant and average velocities, the number of jumps *etc*., can be readily computed from the obtained data. Furthermore, the different flies can be classified by their level of activity (throughout different time periods) or the time they have stayed in a given activity level, or by a given plate region. The average behavior of all the flies in the experiment can also be computed. 

This notable amount of data is useful for analyzing the behavior of the flies in a great number of different experiments.
